# Malaria Diagnosis and Treatment: One Size Does Not Fit All

**DOI:** 10.1371/journal.pmed.0020156

**Published:** 2005-06-28

**Authors:** Chris Drakeley, Roly Gosling, Hugh Reyburn

**Affiliations:** London School of Hygene and Tropical Medicine, London, England, and the Joint Malaria Program, Moshi, Tanzania

Icke and colleagues highlight an online and CD-ROM source available for the teaching of malaria diagnosis [1]. While this is a commendable initiative that provides open access to badly needed training materials, we feel that it is overambitious to propose this Web site (which gives equal weight to diagnosis, treatment, and chemoprophylaxis) as a resource that can be equally relevant to the issues of travel-related malaria, to which the Web site primarily applies, and the problems of malaria diagnosis and treatment in sub-Saharan Africa, which are described as the primary focus in the *PLoS Medicine* article [1].

The microscopic diagnosis of malaria is indeed an important resource in Africa to which the large majority of health facilities do not have access. Even where microscopy is available, there is evidence of substantial overdiagnosis of malaria and that better access to malaria microscopy may not result in better targeting of antimalarials [2,3]. What data there is on the accuracy of slide results suggest that microscope quality, preparation of blood films, and quality of reagents are at least as important in constraining the quality of results as the ability of slide readers to identify Plasmodium parasites from high-quality thin blood films [4–6]. In fact, thick blood films are the norm in Africa, thin films being almost exclusively confined to research, yet these are in the small minority on the Web site.

P. falciparum is overwhelmingly the dominant Plasmodium species seen in Africa, and training guides that try to give a “global overview” run the risk of giving a misleading impression of the relative importance of the four species in any given setting where malaria is endemic. The only actual setting where all species of malaria might be seen relatively frequently would be in travel clinics in developed countries. This point might seem obvious, but for many junior laboratory technicians in Africa hungry for scarce training opportunities it is potentially distracting and confusing.

The problems of simultaneously addressing the issues of malaria in endemic areas of Africa and in travellers from resource-rich countries are particularly applicable to malaria treatment. On the Web site, among the drugs recommended for the treatment of non-severe malaria are quinine, atovaquone/proguanil hydrochloride, mefloquine, and two tetracyclines. In Africa quinine is reserved for severe malaria and is associated with a high rate of failure due to poor adherence in outpatients; atovaquone/proguanil hydrochloride and mefloquine are not widely used because of their high cost; and tetracyclines are contraindicated in children and pregnant women, who bear the overwhelming burden of malaria.

The Web site describes criteria for severe malaria that are not appropriate for Africa. Thus, cerebral malaria, jaundice, renal failure, and lactic acidosis (impossible to detect in most African hospitals where “respiratory distress” is the equivalent criterion) are all listed ahead of severe anaemia, the most common manifestation of severe malaria in Africa. Repeated convulsions are not mentioned, presumably because they are rare among travellers with malaria, but they are common among children in Africa and are associated with a poor outcome. Renal failure as a manifestation of severe malaria is mentioned second but is very uncommon in Africa [7,8].

The apparently large numbers of African health-care workers who have accessed this site might legitimately feel confused by some of these descriptions, and there is no indication of which, if any, sections apply to Africa and which to travellers. While many will interpret the information critically, others may not, especially when English is not their first language and where there is a tendency to accept didactic sources in preference to what is obvious from personal or local experience.

The diagnostic component of the Web site has important potential as a training tool, but we feel that significant modification and more explicit indication of information that is country-specific are needed before its use is likely to result in improved standards of care in African hospitals and clinics.

It is true that Internet technologies can “revolutionise information technology…for healthcare professionals in developing countries”. The World Health Organization and others already have very extensive Web sites that are contributing to this. Searching the Internet on “malaria diagnosis” reveals a number of sites, many with excellent sections, but one is struck by the lack of clarity defining the target audience and context of the problem. For much information available on the Web this may not matter, but for malaria we feel it does.
